# Post-COVID condition or “long COVID”, return-to work, and occupational health research

**DOI:** 10.5271/sjweh.4088

**Published:** 2023-03-30

**Authors:** Alexis Descatha, Bradley A Evanoff, Marc Fadel

**Affiliations:** 1 Univ Angers, CHU Angers, Univ Rennes, Inserm, EHESP, Irset (Institut de recherche en santé, environnement et travail) Angers, UMR_S 1085, SFR ICAT, France; 2 CHU Angers, Poisoning Control Center-Clinical Data Center, Angers, France; 3 Epidemiology and Prevention, Donald and Barbara Zucker School of Medicine, Hofstra Univ Northwell Health, USA; 4 Division of General Medical Sciences, Washington University School of Medicine, St. Louis, MO 13 63310, USA

Since the beginning of the COVID-19 pandemic, there has been a growing literature on COVID and work (1). Major editorials (2–4) and reports from international associations and agencies (5–8) have highlighted the importance of occupational health research in COVID-19 prevention and management. Occupational health research has focused on specific worker populations with high prevalence of COVID-19, particularly healthcare workers, and has studied the associations between different work exposures to SARS-CoV-2 and incidence of COVID-19. Some countries have also begun to provide workers’ compensation for occupationally-acquired COVID-19 (9–13). Return to work (RTW) following COVID-19 is another crucial topic that has been studied among patients with severe cases of SARS-CoV-2 infection in different countries (14–18).

Clinicians have also observed patients who suffer from persistent symptoms following COVID-19, often called “long-COVID” (19). Through analyses of large health databases, a variety of post-acute sequelae among patients with COVID-19 have been identified, including malaise, fatigue, musculoskeletal pain, anemia, and other respiratory, neurocognitive, mental health, metabolic, cardiovascular, and gastrointestinal disorders (20). The World Health Organization defined a “post COVID-19 condition” among individuals with a history of probable or confirmed SARS-CoV-2 infection with symptoms continuing three months from the onset of COVID-19 infection, which last for at least two months, and which cannot be explained by an alternative diagnosis (21). Common symptoms include fatigue, shortness of breath, cognitive dysfunction, and generally have a significant impact on everyday functioning. Symptoms may be of new onset following initial recovery from an acute COVID-19 episode or persist from the initial illness. Symptoms may also fluctuate or relapse over time. The “Global Burden of Disease Long COVID Collaborators”, a worldwide collaboration, defined three main post-COVID condition symptoms: (i) persistent fatigue with bodily pain (myalgia) or mood swings; (ii) cognitive problems, often forgetfulness or concentration difficulties, commonly referred to as “brain fog;” and (iii) ongoing respiratory problems (shortness of breath and persistent cough as the main symptoms) (22). This task force showed that, in a pooled study of over one million subjects, the prevalence of post-COVID condition was 0.9% [95% confidence interval (CI) 0.3–2.0%] after one year, but was at as high as 15% for symptoms that were presents at three months. Different factors were associated with a post-COVID condition, including age 20–49 years, female sex, and initial severity of illness (including hospitalization, intensive care unit admission, and mechanical ventilation) (22–24). For example, 26.6% (95% CI 11.5-47.8) of women who needed intensive care support had a post-COVID condition after a year. In 2023 in the United Kingdom, it was estimated that 2.0 million people living in private households (3.0% of the population) were experiencing self-reported post-COVID conditions (defined as symptoms continuing for more than four weeks after the first confirmed or suspected COVID-19 infection that were not explained by something else) (25). In the sample, more than 85% were in the working age.

The consequences of such unexpected persistence of disease on the health of the workforce are large. Most of the post-COVID symptoms found in the general population were similar for workers: in a recent review including 60 cohort studies of working age adults and 10 cases studies, the most frequently reported post-COVID-19 symptoms were fatigue (92%), shortness of breath (82%), muscle pain (44%) and joint pain (35%) (26). In a recent study with a 15-month follow-up of workers, similar symptoms were reported as well as cognitive symptoms and autonomic dysregulation (27). This study also assessed the Work Ability Index, and found that women had a larger self-reported reduction in work ability following COVID infection than men. RTW and work factors related to post-COVID condition have been examined in small samples of workers, and found that poorer work ability was related to previous comorbidities, symptoms of fatigue, and some occupational and work organizational factors (28–31). Previous comorbidities related to chronic disease (obesity, hypertension and respiratory disorders), were associated with slower RTW among healthcare workers hospitalized for COVID-19. Asthenia / reported loss of memory and sleep disorders were associated with the longest duration of work absence (>3 months) (29). Some type of work and activity sectors have also been related to RTW outcomes. For example, in a small Canadian descriptive study, among healthcare workers physicians had better improvement than nurses and healthcare assistants (28), and business, finance and management sectors had overall the RTW outcome though not significant (31). Modification of work duties improved RTW in this same study, whereas skeptical reactions from employers and colleagues and lack of support from the social welfare system complicated RTW in a qualitative study (30).

Aben et al (32) conducted a study among employees who reported sick due to COVID-19 (N=30 396) or flu-like symptoms not due to COVID-19 (N=15,862), using routinely collected data from a national Dutch occupational health service. Even though there was a 100% RTW rate three months after a flu-like syndrome, the RTW rate after COVID-19 was only 92.8%. The authors were also able to determine important predictors contributing to later return to work in specific statistical models: older age [hazard ratio (HR) 0.99, 95% CI 0.99–0.99], female sex (HR 0.88, 95% CI 0.86–0.90), belonging to a risk group – including chronic illness, compromised immune system, diabetes, and obesity (HR 0.85, 95% CI 0.82–0.89), and some specific symptoms like shortness of breath and fatigue (HR for both symptoms 0.70, 95% CI 0.68–0.72) (32). However, other potential predictors (working conditions and workplace factors, economic context, resources, personal and lifestyle factors) were not exhaustively considered, and understanding causal relation between these factors and the RTW predictors identified is difficult. Aben et al (32) also highlighted that time-to-RTW was shortened as different virus variants became dominant over time. For example, 12.8% of employees who contracted COVID-19 were absent from work for >12 weeks during the alpha virus dominant period, but this number declined to 5.8% in the delta virus-dominant period and to 1.4% in the omicron virus dominant period (32).

Strategies promoting return to work for those with post-COVID-19 conditions will need to be implemented and could be comparable to programs developed for other chronic conditions, since post-COVID conditions share some similarities to post-intensive care syndrome, fibromyalgia, and chronic fatigue syndrome (15, 33, 34). These patients have better outcomes following integrative health approach that combines traditional medical management, non-pharmacological treatments including physical therapy, and behavior and lifestyle changes (35, 36). As with RTW following other illnesses, RTW efforts post-COVID should look out for potential red flags or complications (19). Guidelines recommend that occupational practitioners should be included in the process as early as possible, and give importance to job accommodations for improving work ability of such workers (5, 8, 37). An observational study on a small sample of workers undergoing specific rehabilitation for post-COVID conditions reported significant but modest improvements on a variety of symptoms, though only half of the participants were able to return to work (31). Additional research in working populations is needed with larger observational cohorts, randomized controlled trials, and mixed approaches to evaluate the cost-effectiveness of integrative care and other approaches in preserving work ability for these patients (38–40).

In conclusion, COVID-19 remains an important topic for the occupational health research agenda, including acute and post COVID conditions. Although there is still debate about the definition of what a ‘post-COVID condition’ entails, the sheer number of patients who are not returning to work in a timely manner or returning to work with limitations, and the lack of research interventions available should lead occupational health practitioners and researchers to work not only to prevent infection but to prevent or reduce work disability resulting from the COVID-19 pandemic and future pandemics.

## References

[1] Burdorf A, Porru F, Rugulies R (2021). The COVID-19 pandemic:one year later - an occupational perspective. Scand J Work Environ Health.

[2] Burdorf A, Porru F, Rugulies R (2020). The COVID-19 (Coronavirus) pandemic:consequences for occupational health. Scand J Work Environ Health.

[3] Sim MR (2020). The COVID-19 pandemic:major risks to healthcare and other workers on the front line. Occup Environ Med.

[4] Descatha A (2020). [COVID-19:Tribute to Health Care Warriors, to their occupational health units, and to their strategists. Arch. Mal. Prof. Environ.

[5] Global impact and policy recommendations (COVID-19 and the world of work) [Internet].

[6] COVID-19:Occupational health and safety for health workers:interim guidance 2 February 2021 [Internet].

[7] COVID-19 - Control and Prevention |Occupational Safety and Health Administration [Internet].

[8] COVID-19:Back to the workplace - Adapting workplaces and protecting workers |Safety and health at work EU-OSHA [Internet].

[9] Descatha A, Fadel M, Pitet S, Verdun-Esquer C, Esquirol Y, Legeay C (2021). SARS-CoV-2 (COVID-19) Job Exposure Matrix:“Mat-O-Covid”Creation (COVID-Mate in French), accuracy study, and perspectives. Archives des Maladies Professionnelles et de l'Environnement.

[10] Oude Hengel KM, Burdorf A, Pronk A, Schlünssen V, Stokholm ZA, Kolstad HA (2022). Exposure to a SARS-CoV-2 infection at work:development of an international job exposure matrix (COVID-19-JEM). Scand J Work Environ Health.

[11] Descatha A, Sembajwe G, Gilbert F, Fadel M, Mat-O-Covid Investigation Group null (2022). Mat-O-Covid:Validation of a SARS-CoV-2 Job Exposure Matrix (JEM) Using Data from a National Compensation System for Occupational COVID-19. Int J Environ Res Public Health.

[12] van der Feltz S, Peters S, Pronk A, Schlünssen V, Stokholm ZA, Kolstad HA (2023). Validation of a COVID-19 Job Exposure Matrix (COVID-19-JEM) for Occupational Risk of a SARS-CoV-2 Infection at Work:Using Data of Dutch Workers. Ann Work Expo Health.

[13] Fadel M, Gilbert F, Legeay C, Dubée V, Esquirol Y, Verdun-Esquer C (2022). Association between COVID-19 infection and work exposure assessed by the Mat-O-Covid job exposure matrix in the CONSTANCES cohort. Occup Environ Med.

[14] Hodgson CL, Higgins AM, Bailey MJ, Mather AM, Beach L, Bellomo R (2021). The impact of COVID-19 critical illness on new disability, functional outcomes and return to work at 6 months:a prospective cohort study. Crit Care.

[15] Godeau D, Petit A, Richard I, Roquelaure Y, Descatha A (2021). Return-to-work, disabilities and occupational health in the age of COVID-19. Scand J Work Environ Health.

[16] Gualano MR, Rossi MF, Borrelli I, Santoro PE, Amantea C, Daniele A (2022). Returning to work and the impact of post COVID-19 condition:A systematic review. Work.

[17] Griffiths D, Sheehan L, van Vreden C, Petrie D, Whiteford P, Sim MR (2022). Changes in work and health of Australians during the COVID-19 pandemic:a longitudinal cohort study. BMC Public Health.

[18] Fourie M, van Aswegen H (2022). Outcome of survivors of COVID-19 in the intermediate phase of recovery:A case report. S Afr J Physiother.

[19] DeMars J, Brown DA, Angelidis I, Jones F, McGuire F, O'Brien KK (2022). What is Safe Long COVID Rehabilitation?. J Occup Rehabil.

[20] Al-Aly Z, Xie Y, Bowe B (2021). High-dimensional characterization of post-acute sequelae of COVID-19. Nature.

[21] A clinical case definition of post COVID-19 condition by a Delphi consensus 6 October 2021 [Internet].

[22] Wulf Hanson S, Abbafati C, Aerts JG, Al-Aly Z, Ashbaugh C, Global Burden of Disease Long COVID Collaborators (2022). Estimated Global Proportions of Individuals With Persistent Fatigue, Cognitive, and Respiratory Symptom Clusters Following Symptomatic COVID-19 in 2020 and 2021. JAMA.

[23] Joshee S, Vatti N, Chang C (2022). Long-Term Effects of COVID-19. Mayo Clin Proc.

[24] Wulf Hanson S, Abbafati C, Aerts JG, Al-Aly Z, Ashbaugh C, Ballouz T (2022). A global systematic analysis of the occurrence, severity, and recovery pattern of long COVID in 2020 and 2021. medRxiv.

[25] Prevalence of ongoing symptoms following coronavirus (COVID-19) infection in the UK - Office for National Statistics [Internet].

[26] Kokolevich ZM, Crowe M, Mendez D, Biros E, Reznik JE (2022). Most Common Long COVID Physical Symptoms in Working Age Adults Who Experienced Mild COVID-19 Infection:A Scoping Review. Healthcare (Basel).

[27] Sansone D, Tassinari A, Valentinotti R, Kontogiannis D, Ronchese F, Centonze S (2022). Persistence of Symptoms 15 Months since COVID-19 Diagnosis:Prevalence, Risk Factors and Residual Work Ability. Life (Basel).

[28] Grazzini M, Lulli LG, Mucci N, Paolini D, Baldassarre A, Gallinoro V (2022). Return to Work of Healthcare Workers after SARS-CoV-2 Infection:Determinants of Physical and Mental Health. Int J Environ Res Public Health.

[29] Mendola M, Leoni M, Cozzi Y, Manzari A, Tonelli F, Metruccio F (2022). Long-term COVID symptoms, work ability and fitness to work in healthcare workers hospitalized for Sars-CoV-2 infection. Med Lav.

[30] Kohn L, Dauvrin M, Detollenaere J, Primus-de Jong C, Maertens de Noordhout C, Castanares-Zapatero D (2022). Long COVID and return to work:a qualitative study. Occup Med (Lond).

[31] Brehon K, Niemeläinen R, Hall M, Bostick GP, Brown CA, Wieler M (2022). Return-to-Work Following Occupational Rehabilitation for Long COVID:Descriptive Cohort Study. JMIR Rehabil Assist Technol.

[32] Aben B, Kok RN, de Wind A (2023). Return-to-work rates and predictors of absence duration after COVID-19 over the course of the pandemic. Scand J Work Environ Health.

[33] Dotan A, David P, Arnheim D, Shoenfeld Y (2022). The autonomic aspects of the post-COVID19 syndrome. Autoimmun Rev.

[34] Uchiyama Y, Sasanuma N, Nanto T, Fujita K, Takahashi M, Iwasa S (2021). COVID-19 Patient Returned to Work after Long Hospitalization and Follow-up:A Case Report. Prog Rehabil Med.

[35] Roth A, Chan PS, Jonas W (2021). Addressing the Long COVID Crisis:Integrative Health and Long COVID. Glob Adv Health Med.

[36] Paz LES, Bezerra BJ, da S, Pereira TM, de M, da Silva WE (2021). COVID-19:the importance of physical therapy in the recovery of workers'health. Rev Bras Med Trab.

[37] Clin B, Esquirol Y, Gehanno JF, Letheux C, Gonzalez M, Pairon JC (2021). Rôle des services de santéau travail dans le repérage et l'accompagnement des personnes concernées par des symptômes persistants suite àla Covid-19. Recommandations de la Sociétéfrançaise de médecine du travail (SFMT). Archives des Maladies Professionnelles et de l'Environnement.

[38] Müller K, Zwingmann K, Auerswald T, Berger I, Thomas A, Schultz AL (2021). Rehabilitation and Return-to-Work of Patients Acquiring COVID-19 in the Workplace:A Study Protocol for an Observational Cohort Study. Front Rehabil Sci.

[39] do Prado CB, Emerick GS, Cevolani Pires LB, Salaroli LB (2022). Impact of long-term COVID on workers:A systematic review protocol. PLoS One.

[40] Sivan M, Greenhalgh T, Darbyshire JL, Mir G, O'Connor RJ, Dawes H (2022). LOng COvid Multidisciplinary consortium Optimising Treatments and servIces acrOss the NHS (LOCOMOTION):protocol for a mixed-methods study in the UK. BMJ Open.

[41] Alkodaymi MS, Omrani OA, Fawzy NA, Shaar BA, Almamlouk R, Riaz M (2022). Prevalence of post-acute COVID-19 syndrome symptoms at different follow-up periods:a systematic review and meta-analysis. Clin Microbiol Infect.

[42] Valter R, Sembajwe G, Descatha A, Fadel M (2022). Comparison of different estimators of SARS-CoV-2 pandemic activity on geographical and temporal levels. Front Public Health.

